# Oat Bran Hydrolysates Alleviate Oxidative Stress and Inflammation in d‐Galactose‐Induced Aging Mice

**DOI:** 10.1002/fsn3.70433

**Published:** 2025-06-17

**Authors:** Haoyuan Ma, Rui Wang, Minjun Sun, Sarina Ma, Meili Zhang

**Affiliations:** ^1^ College of Food Science and Engineering Inner Mongolia Agricultural University Hohhot China

**Keywords:** aging, gut microbiota, hydrolysates, metabolomics, oat bran, oxidative stress

## Abstract

Oat bran hydrolysates exhibit various biological activities, including antioxidant and immunomodulation activities. Enzymatic hydrolysis can improve the antioxidant capacity of oat bran protein. In this study, Alcalase was used to hydrolyze oat bran protein and the resulting hydrolysates were separated by ultrafiltration, the antioxidant activity of fraction F1 (molecular weight > 10 kDa, OBH) was the highest in the four fractions, and selected for exploring the functional characteristics: d‐galactose (d‐gal; 300 mg/kg/day) induced aging of CL57BL/6J mice and OBH (200, 400, 800 mg/kg/day) intervened for 8 weeks. Normal mice were compared with aging mice induced by d‐gal and aged mice treated with OBH. OBH intervention significantly improved the antioxidant oxidase activities in aging mice, such as total antioxidant capacity, catalase and glutathione peroxidase activities; it decreased malondialdehyde levels, and reduced the levels of tumor necrosis factor‐α, interleukin (IL)‐1β and IL‐6 in the brain and serum. Pathological observations showed that OBH prevented brain damage. The results of high‐throughput sequencing showed that the relative abundance of *Verrucomicrobiota* was decreased, whereas *Duncaniella*, *Paramuribaculum*, *Odoribacter*, and *Alistipes_A* were increased. Metabolomics analysis showed that OBH mainly altered glycerophospholipid, cysteine and methionine metabolism. These results indicated that OBH has great potential as a functional food, which can alleviate oxidative damage and inflammation in the serum and brain, maintain the stability of intestinal bacteria, alleviate metabolic disorders and delay aging.

## Graphical abstract

The oat bran hydrolysate obtained after enzymatic hydrolysis can alleviate oxidative stress and inflammation in d‐galactose‐induced aged mice.

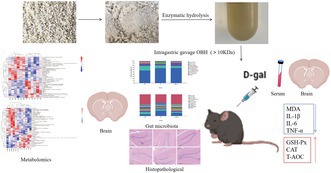

**Abbreviations**
CAT: catalase
d‐gal: d‐galactose
GSH‐Px: glutathione peroxidase
IL‐1β: interleukin (IL)‐1β
IL‐6: interleukin (IL)‐6
MDA: malondialdehyde
OBH: Oat bran hydrolysate
ROS: reactive oxygen species
T‐AOC: total antioxidant activity
TNF‐α: tumor necrosis factor‐α

## 1 Introduction

Aging represents an extremely complex multifactorial natural process, with the typical feature of deterioration of body functions, which includes a gradual decline in antioxidant function (Forman 2016; Galkin et al. 2019). The World Health Organization estimates that the proportion of the global population aged ≥ 60 years may reach 2.1 billion in 2050, and with increased aging population, anti‐aging emerges as a critical problem (Wyss‐Coray 2016). Oxidative stress plays a key role in inducing aging and results from harmful attacks of free radicals on cells or intercellular contents (mostly reactive oxygen species [ROS]) (Viña 2019). Excessive free radical production or accumulation disrupt the redox balance, which generates oxidative stress, causes oxidative damage to cells and biomolecules, and leads to the development of different disorders, like Alzheimer's disease, cancers, and cardiovascular diseases (Abdi and Ali 1999; Liu et al. 2024). Nowadays, it has become an inevitable trend to reduce oxidative stress and delay aging by consuming foods with antioxidant activity.

Oat bran is a by‐product of oat processing and has a high protein content (13%–20%) (Guan et al. 2018). Oat bran hydrolysates are bioactive and have positive health effects, such as antioxidant, lipid‐lowering, and antidiabetic activities (Baakdah and Tsopmo 2016; Campos Espinosa et al. 2022). Esfandi et al. (2019) reported that the enzymatic hydrolysis of oat bran proteins yielded hydrolysates that can affect the antioxidative stress capacity of hepatocytes, and these hydrolysates prevented oxidative stress‐induced cell death. Some studies have shown good free radical scavenging activity of oat bran proteolytic products (Walters et al. 2020). However, the studies mainly focused on the in vitro antioxidant capacity of oat bran hydrolysates; it is hypothesized that oat bran hydrolysates have an inhibitory effect on oxidative stress in vivo, and there are few data on the effectiveness of oat bran hydrolysates in regulating oxidative stress in vivo through antioxidant enzyme activity, regulation of gut microbes, and metabolites.

d‐galactose (d‐gal) represents the natural reducing sugar, which can be converted to glucose in the body for metabolism (Zhao et al. 2021). Nonetheless, high d‐gal concentrations are metabolized to galactitol and hydrogen peroxide, which eventually generate ROS, and increased ROS levels cause oxidative stress, mitochondrial dysfunction, and inflammation within different mammalian organs, which ultimately lead to cellular apoptosis (Xu et al. 2016). d‐gal induced aging in mice shows close symptoms to natural aging; thus, mouse models are widely adopted for studying aging pharmacology (Haider et al. 2015). However, as we have seen yet, there are few reports on the protective effect of OBH on the brain of aging mice.

Therefore, the purpose was to study the potential role of OBH, which is selected from ultrafiltration with the strongest antioxidant activity, in alleviating oxidative stress and inflammation in brain tissue, determining physiological parameters, oxidative stress, and inflammation levels in d‐gal‐induced aging mice. In addition, in order to study the possible alleviation effect of OBH, the gut microbiota and brain metabonomics of aged mice induced by d‐gal were analyzed. The aim was to evaluate the functionality of OBH, provide a theoretical basis for an in‐depth study of OBH in vivo, and provide suggestions for developing nutritious food with anti‐aging function.

## 2 Materials and Methods

### 2.1 Chemicals and Reagents

Oat bran was acquired from Inner Mongolia Xibei Huitong Agricultural Science and Technology Development Co. Ltd. (Inner Mongolia, China). Ultrafiltration tubes were purchased from PALL (USA). Trolox, vitamin C (VC), and d‐gal were provided by Dalian Meilun Biotechnology Co. Ltd. (Dalian, China). A045‐2 Total protein content, A003‐1 malondialdehyde (MDA), A007‐1 catalase (CAT), A005‐1 glutathione peroxidase (GSH‐Px), and A015‐1 total antioxidant capacity (T‐AOC) assay kits were provided by Nanjing Jiancheng Institute of Biological Engineering (Nanjing, China), and hydrochloric acid, sodium hydroxide, and petroleum ether of analytical grade were used.

### 2.2 Preparation of OBH

Oat bran coarse powder was ground and passed through an 80 mesh sieve, defatted by petroleum ether, then dissolved in water at 1:20 (w/v) material‐liquid ratio, adjusting the pH to 9.5 using 0.5 M NaOH, immersed in a 50°C water bath, and stirred for 1 h and then centrifuged. 1 M HCl was added to the supernatant until the pH was adjusted to 4.0, which is the isoelectric precipitation point. The mixture was left still for 1 h and centrifuged. After dissolution in deionized water for further homogenization, the precipitate was subjected to freeze‐drying to obtain oat bran protein. The hydrolysis was carried out with Alcalase (15,000 U/g) at a substrate concentration of 2% (w/v) at 50°C and pH 9.5 for 3 h. The mixed solution was kept for a 10‐min period in a boiling water bath to inactivate the enzyme. The solution was later cooled to ambient temperature, adjusted to pH 4.0, left still for a 30‐min duration, and centrifuged (14,000 *g*, 20 min, 4°C) to obtain supernatants containing oat bran protein hydrolysates, which were freeze‐dried. Finally, the powder was stored at −20°C prior to use. Ultrapure water was later added to dissolve oat bran protein hydrolysates at a certain concentration and sequentially ultrafiltered using ultrafiltration membranes with retention capacities of 10, 3, and 1 kDa. Four fractions (F1–F4) with molecular weights > 10, 3–10, 1–3, and < 1 kDa separately were collected, freeze‐dried, then dissolved in ultrapure water at 10 mg/mL to assess the antioxidant capacity of each fraction.

### 2.3 Antioxidant Activity Assay

#### 2.3.1 DPPH Free‐Radical Scavenging Rate

The DPPH approach was utilized to measure free‐radical scavenging capacity, referencing Magangana et al. (2021) methods with slight modification. Briefly, the diluted sample (10 μL) was added to the DPPH solution (190 μL, 60 μM/L) in a 96‐well plate and kept for 1 h in the dark at 30°C. Absorbance values were detected at 515 nm using microplate readers. The standard curve was generated using 100–1500 μmol/L Trolox, and the standard curve equation was as follows: *y* = 0.0493*x* + 9.9268 (*R* ^2^ = 0.9936). The DPPH· scavenging rate was determined using the standard curve, which was indicated by the mass of Trolox/g dry mass sample (μmol/g) according to Equation (1) as shown below:

(1) $$Y/\%=1-A_{1}/A_{0}\times 100\%$$

where *Y* is DPPH· scavenging rate (%) by sample or Trolox, *A*_1_ stands for sample or Trolox absorbance, while *A*_0_ represents blank control absorbance.

#### 2.3.2 Determination of ABTS ^+^ Scavenging Rate

We carried out the ABTS assay as described by Setti et al. (2020) after slight modifications. To prepare the ABTS stock solution, 100 mL (7 mM) of ABTS was mixed with 100 mL potassium persulfate (2.45 mM) and incubated for 14 h at 30°C in the dark. The ABTS stock was diluted with phosphate‐buffered saline buffer (0.05 M, pH 7.4) until reaching the 0.7 ± 0.02 absorbance value at 734 nm to obtain the ABTS working solution. The sample (10 μL) was mixed with the ABTS cation radical solution (190 μL) within the 96‐well plate, allowing it to react at 25°C for 5 min in the dark. Then, the microplate reader was utilized to measure the absorbance at 734 nm. The standard curve was generated for 100–1000 μmol/L Trolox, and the standard curve equation was shown below: *y* = 0.0214*x* + 4.5768 (*R* ^2^ = 0.9959). The calculation method was the same as Equation (1), which was indicated by the mass of Trolox/g dry mass (μmol/g).

#### 2.3.3 Determination of Ferric Iron Reduction Ability

We analyzed the ferric iron reduction ability as described by Samaei et al. (2021) with some modifications. To begin, 200 μL of the sample solution was added to a reaction tube (dilution may be necessary) with the addition of 6 mL of the FRAP working solution (consisting of 0.3 M/L (pH 3.6)) acetate buffer, 20 mM/L ferric chloride (FeCl_3_) solution, and 10 mM/L of TPTZ solution at the 10:1:1 ratio; the reagent was prepared freshly daily and 600 μL of distilled water. The solution was mixed well and left for 10 min at 37°C for reaction. The absorbance values were taken at 593 nm. The sample was replaced with a standard solution of FeSO_4_ (50–250 μmol/L) to create the standard curve, and the standard curve equation was as follows: *y* = 7.174*x* − 0.0329 (*R* ^2^ = 0.9996), and the results were expressed as Fe^2+^ concentration (μmol/L).

### 2.4 Chemical Composition and Amino Acid Composition of OBH

The protein content was determined using the Kjeldahl method with a conversion factor of 6.25 (AOAC 1995), and the lipid, moisture, starch, and amino acid contents were analyzed according to the National Standards of China (GB 5009.6–2016, GB 5009.3–2016, GB 5009.9–2016, GB 5009.124–2016, respectively).

### 2.5 Animals and Experiments

A total of sixty 6–8‐week‐old SPF grade male CL57BL/6J mice were purchased from Sipeifu (Beijing) Biotechnology Co. Ltd. (License No. SYXK (Jing) 2019‐0030). They were housed at 22 ± 2°C, with humidity level at 50 ± 5% humidity, and 12‐h/12‐h light and dark cycle. All doses of d‐galactose and OBH, the experimental period we used was determined according to Zhang, He, et al. (2020). After 1 week of acclimatization feeding, the mice were randomized into six groups (*n* = 10 each): normal (normal diet), d‐gal‐induced aging model (model); VC‐positive control (VC); low‐, medium‐, and high‐dose OBH groups (Table 1). The mice in the model, VC, and OBH dose groups were given subcutaneous injections of 300 mg/kg d‐gal, while those in the normal group were given the equivalent amount of normal saline. The mice in the three OBH treated groups were gavaged daily with 200, 400, and 800 mg/kg of OBH, the dosage for those in the VC group was 100 mg/kg, and the normal and model groups of mice were gavaged with sterile water. All the mice were gavaged and injected at regular intervals daily, and changes in their body weights and food intake were recorded weekly for 8 weeks. Fasting for 12 h after the final gavage, after weighing the mice, all mice were anesthetized with isoflurane, blood was collected through eye socket bleeding, and the mice were euthanized by cervical dislocation. The liver and brain tissues were quickly taken out, washed with normal saline and weighed. The organ index was calculated by dividing the organ weight (mg) by the body weight (g). The animal experimental protocols were approved by the Laboratory Animal Welfare and Ethics Committee of Inner Mongolia Agricultural University.

**TABLE 1:** Animal grouping and management.

Group	1–8 week	
	Subcutaneous injection	Intragastrical
Normol diet	0.9% Physiological saline (0.1 mL)	Sterile water (0.1 mL)
Model	d‐gal (300 mg/kg/day, 0.1 mL)	Sterile water (0.1 mL)
VC	d‐gal (300 mg/kg/day, 0.1 mL)	VC (100 mg/kg/day, 0.1 mL)
OBH‐L	d‐gal (300 mg/kg/day, 0.1 mL)	OBP (200 mg/kg/day, 0.1 mL)
OBH‐M	d‐gal (300 mg/kg/day, 0.1 mL)	OBP (400 mg/kg/day, 0.1 mL)
OBH‐H	d‐gal (300 mg/kg/day, 0.1 mL)	OBP (800 mg/kg/day, 0.1 mL)

### 2.6 Histopathology of the Brain

The brain tissues of the mice were immediately fixed with 4% paraformaldehyde for a 24‐h period. The tissues were dehydrated with gradient alcohol prior to paraffin embedding and slicing in 4‐μm sections and hematoxylin and eosin (H&E) staining. Pathological images were acquired using the light microscope (Nikon Eclipse CI) to determine histopathological alterations.

### 2.7 Antioxidant Oxidase Activities Within the Serum and Brain Tissues

The blood samples were extracted through eyeball removal, then centrifuged (3000 r/min, 4°C) for a 10‐min. The serum was collected to examine the biochemical indices. Saline was added to the brain tissues at the ratio of 1:9 (w/v), which were sheared and a homogenate was prepared within the ice‐water bath, followed by 15‐min centrifugation at 3000 r/min to obtain the supernatant.

Total protein kit was used to determine the sample's protein concentration (Jiancheng, Nanjing, China). The activities of antioxidase activities such as GSH‐Px, CAT, and T‐AOC, and MDA contents in the serum and brain homogenates were assessed in commercial biochemical kits (Jiancheng, Nanjing, China). According to the manufacturer's instructions added the reagent, then centrifuged the supernatant to detect the absorbance value; the antioxidant enzyme activities were calculated according to the formula.

### 2.8 Determination of Cellular Immune Factors in Serum and Brain Tissue

ELISA kits from Jingmei (Jiangsu, China) were employed to quantify levels of TNF‐α, IL‐1β, and IL‐6 in the serum and brain tissues of aged mice. 50 μL of different concentrations of standards was added to each standard well of the strip, and 10 μL of the samples to be tested was added to the sample wells, followed by the addition of 40 μL of sample diluent. Subsequently, 100 μL of horseradish peroxidase (HRP)‐labeled detection antibody was dispensed into each well. The plate was sealed and incubated at 37°C for 60 min. Afterward, the plate underwent five washes before adding substrates A and B (50 μL/well). The plate was performed for 15 min at 37°C in darkness, after which a stopping solution was added to halt the reaction. Finally, optical density (OD) values were recorded at 450 nm within 15 min using a plate reader, whereas sample concentrations were determined by referencing a constructed curve equation.

### 2.9 16S rRNA High‐Throughput Gene Sequencing

Sterile test tubes were used to collected fresh mouse feces, and liquid nitrogen was used for freezing. After the DAN was extracted from the feces, the total DNA quantity and quality were analyzed with Nanodrop and 1.2% agarose gel electrophoresis respectively. The Illumina's TruSeq Nano DNA LT Library Prep Kit was used for preparing sequencing libraries.

The microbial composition and diversity of the fecal samples were analyzed through bioinformatics using the Personal Genescloud Platform.

### 2.10 Brain Tissue Differential Metabolite Assay

After the mouse brain tissue samples were thawed slowly at 4°C, 25 mg of the brain tissues were taken and added into the pre‐chilled methanol/acetonitrile/water solution (500 μL, 2:2:1, v/v), vortexed and mixed for 30 s. Homogenized (35 Hz, 4 min) the sample and then proceeded to ice bath ultrasonic treatment for 5 min, and repeated the proceeding steps three times. The mixtures were kept at −40°C for 1 h, followed by 20 min centrifugation (14,000 *g*, 4°C). The Vanquish (Thermo Fisher Scientific) ultra‐high‐performance liquid chromatograph was utilized to detect supernatants.

Proteo Wizard software was employed for converting raw data into mzXML format, whereas the R package was adopted for metabolite identification followed by visualization.

### 2.11 Statistical Analysis

Conducted all experiments in triplicate, and mean ± standard deviation (SD) was used to indicate experiment data. Analysis of variance (ANOVA) and *t*‐test statistics were performed by SPSS 25.0 (IBM, USA), and graphed by GraphPad Prism 8.2.0 (GraphPad Software, USA). Results were considered significant when *p* < 0.05.

## 3 Results and Discussion

### 3.1 Antioxidant Effects of Ultrafiltration Fractions

**FIGURE 1:**
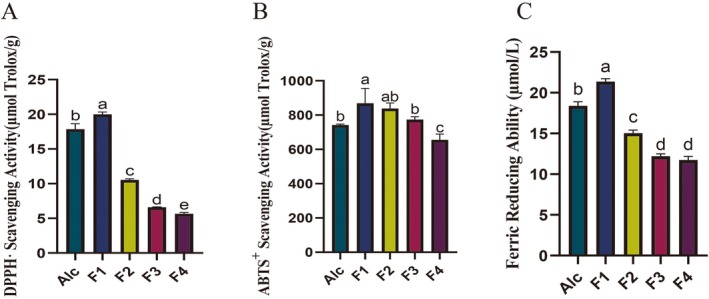
Antioxidant activities of Alcalase hydrolysate (Alc), fractions F1 (> 10 kDa), F2 (3–10 kDa), F3 (1–3 kDa), and F4 (< 1 kDa) of oat bran proteins hydrolyzed by Alcalase. (A) DPPH· scavenging activity; (B) ABTS^+^ scavenging activity; (C) ferric ion reducing ability. Significant (*p* < 0.05) differences in antioxidant activity between groups were indicated by different lowercase letters (a–e).

The antioxidant activities of Alcalase hydrolysate (Alc) and fractions F1–F4 were comprehensively assessed by performing three different assays, namely DPPH· and ABTS^+^ scavenging abilities, and ferrous ion reduction capacity. Further investigation was conducted on the fraction which had the highest antioxidant activity (Figure 1).

The antioxidant effect of the Alcalase hydrolysate (Alc) and ultrafiltration separation fractions were determined (Figure 1). As shown in Figure 1, the DPPH· scavenging rates of ultrafiltration fractions were in descending order of F1 > Alc>F2 > F3 > F4, and the F1 was the most potent in scavenging DPPH·, with a scavenging rate of 19.83 ± 0.15 μmol Trolox/g. The differences in DPPH· scavenging rates among the fractions could be associated with different molecular weights and amino acid compositions in hydrolysates and peptide fractions (Bamdad et al. 2011). Similarly, Farvin et al. (2010) found that the high‐molecular‐weight hydrolysates (> 30 and 10 – 30 kDa) exhibited higher DPPH· scavenging activity than the lower‐molecular‐weight counterparts did. The trend of ferric ion‐reducing ability was identical to that of DPPH· scavenging, with F1 (21.36 ± 0.35 μmol/L) showing significantly stronger ferric ion‐reducing ability than Alc and the remaining fractions (*p* < 0.05), whereas F3 and F4 showed weaker ferric ion‐reducing ability (12.21 ± 0.24 μmol/L and 11.74 ± 0.35 μmol/L), with no significant differences. The ABTS^+^ scavenging rates of F1 (868.57 ± 87.01 μmol Trolox/g) and F2 (814.84 ± 32.48 μmol Trolox/g) were not significantly different but both of them exhibited greater effect than those of the other fractions probably due to the most of the compounds with the ability of scavenging ABTS^+^ present in F1 and F2. F1 (> 10 kDa, OBH) obtained after ultrafiltration separation exhibited the highest ABTS^+^ and DPPH· scavenging capacity as well as ferric ion reduction than the other fractions did. Yeşiltaş et al. (2023) suggested that compounds with high molecular weights exhibited high antioxidant effects after the classification of potato protein hydrolysates obtained by ultrafiltration. Wu et al. (2016) analyzed the antioxidant properties of protein hydrolysates obtained in Douchi through membrane ultrafiltration and showed that those with higher molecular weights exhibited higher antioxidant activity. Therefore, F1 (OBH) was selected to treat aging mice to investigate OBH inhibition against oxidative stress.

### 3.2 Chemical Composition and Amino Acid Composition of OBH

#### 3.2.1 Chemical Composition of OBH

The chemical composition of the hydrolysis mixture was determined: protein (66.48 ± 1.36 g/100 g), starch (5.52 ± 0.28 g/100 g), moisture (0.89 ± 0.10 g/100 g), and lipid (1.52 ± 0.09 g/100 g). The yield of the OBH was 54.2 ± 1.87%.

#### 3.2.2 Amino Acid Composition of OBH

The amino acid composition of the oat bran hydrolysate was presented in Table 2, to identify specific amino acids that may contribute to its antioxidant properties. The hydrolysates consist of 17 amino acids, including seven essential amino acids for humans (valine, isoleucine, leucine, phenylalanine, methionine, threonine, and lysine), constituting 32.60% of the total amino acid content. Notably, glutamic acid (119.09 mg/g), aspartic acid (37.09 mg/g), leucine (37.47 mg/g), arginine (33.38 mg/g), and phenylalanine (30.81 mg/g) were identified as the predominant amino acids in OBH. Glutamic acid and aspartic acid were acidic amino acids whereas arginine were basic amino acids, it has been reported that acidic and basic amino acid residues play a key role in chelating iron ions. Basic amino acids act as hydrogen donors, while acidic amino acids contribute electrons during free‐radical interactions, enhancing antioxidant capacity (Xu et al. 2023; Wang et al. 2022). Additionally, phenylalanine, an aromatic amino acid, exhibits potent free radical scavenging activity due to its aromatic structure and phenolic groups (Nimalaratne et al. 2011). These findings collectively indicate that there may be a strong correlation between the amino acid composition of OBH and its antioxidant activity.

**TABLE 2:** Amino acid composition of oat bran hydrolysates.

Amino acids	Concentration/(mg/g)
Aspartic acid	37.09 ± 0.41
Threonine	16.47 ± 0.27
Serine	22.34 ± 0.75
Glutamic acid	119.09 ± 0.83
Glycine	21.55 ± 0.36
Alanine	22.89 ± 0.33
Cysteine	6.57 ± 0.13
Valine	26.56 ± 0.06
Methionine	7.56 ± 0.03
Isoleucine	18.66 ± 0.19
Leucine	37.47 ± 0.27
Tyrosine	18.82 ± 0.07
Phenylalanine	30.81 ± 4.19
Lysine	16.87 ± 0.18
Histidine	10.76 ± 0.25
Arginine	33.38 ± 0.12
Proline	26.67 ± 0.41
Total essential amino acids	154.39
Total amino acids	473.55

### 3.3 Changes in Body Weight, Organ Index and Food Intake in Mice

In order to analyze how OBH affected aging mice, we examined the changes in their body weights, organ index, and food intake (Table 3a–c). We found that the differences in body weights, organ index, and food intake did not show significant differences among normal, d‐gal‐induced aging, and OBH‐intervened group mice (Zou et al. 2023).

**TABLE 3:** (a) Effect of OBH on body weight in mice (*n* = 10). (b) Effect of OBH on organ index in mice (*n* = 10). (c) Effect of OBH on food intake in mice (*n* = 10).

(a)			
Group	Body weight (g)		
	Initial weight	Weight after experiment	Experimental weight gain
Normol diet	19.41 ± 0.73	24.06 ± 1.91	4.80 ± 1.57
Model	19.41 ± 1.00	23.87 ± 1.52	4.83 ± 0.96
VC	19.46 ± 0.48	22.80 ± 0.90	3.94 ± 0.73
OBH‐L	19.77 ± 0.50	23.77 ± 1.28	4.23 ± 1.26
OBH‐M	19.42 ± 0.69	23.58 ± 0.78	4.47 ± 0.67
OBH‐H	19.00 ± 0.83	23.28 ± 1.72	4.81 ± 1.61

(b)		
Group	Liver index (mg/g)	Brain index (mg/g)
Normol diet	36.85 ± 3.29	16.88 ± 0.94
Model	34.96 ± 13.69	15.99 ± 0.85
VC	35.84 ± 4.90	17.46 ± 0.78
OBH‐L	30.62 ± 4.04	17.13 ± 0.64
OBH‐M	32.83 ± 2.98	17.31 ± 0.90
OBH‐H	19.00 ± 0.83	17.51 ± 0.99

(c)						
Time	Food intake (g/mouse/day)					
	Normol diet	Model	VC	OBH‐L	OBH‐M	OBH‐H
1st week	3.40 ± 0.17	3.40 ± 0.08	3.36 ± 0.11	3.34 ± 0.10	3.41 ± 0.12	3.36 ± 0.21
2nd week	3.72 ± 0.18	3.74 ± 0.13	3.70 ± 0.11	3.60 ± 0.05	3.59 ± 0.16	3.58 ± 0.14
3rd week	3.91 ± 0.17	4.07 ± 0.16	3.67 ± 0.13	3.83 ± 0.06	3.71 ± 0.05	3.68 ± 0.36
4th week	3.88 ± 0.12	3.99 ± 0.27	3.47 ± 0.13	3.78 ± 0.01	3.57 ± 0.20	3.70 ± 0.41
5th week	4.05 ± 0.07	4.41 ± 0.24	3.74 ± 0.09	4.22 ± 0.02	4.02 ± 0.13	4.29 ± 0.33
6th week	3.82 ± 0.04	4.17 ± 0.27	3.66 ± 0.11	4.05 ± 0.12	3.82 ± 0.20	4.05 ± 0.37
7th week	3.78 ± 0.28	4.13 ± 0.27	3.63 ± 0.01	3.90 ± 0.19	3.81 ± 0.14	4.08 ± 0.57
8th week	3.93 ± 0.12	4.19 ± 0.40	3.67 ± 0.05	4.08 ± 0.26	3.91 ± 0.31	3.92 ± 0.62

### 3.4 Analysis of Histopathological Alterations

**FIGURE 2:**
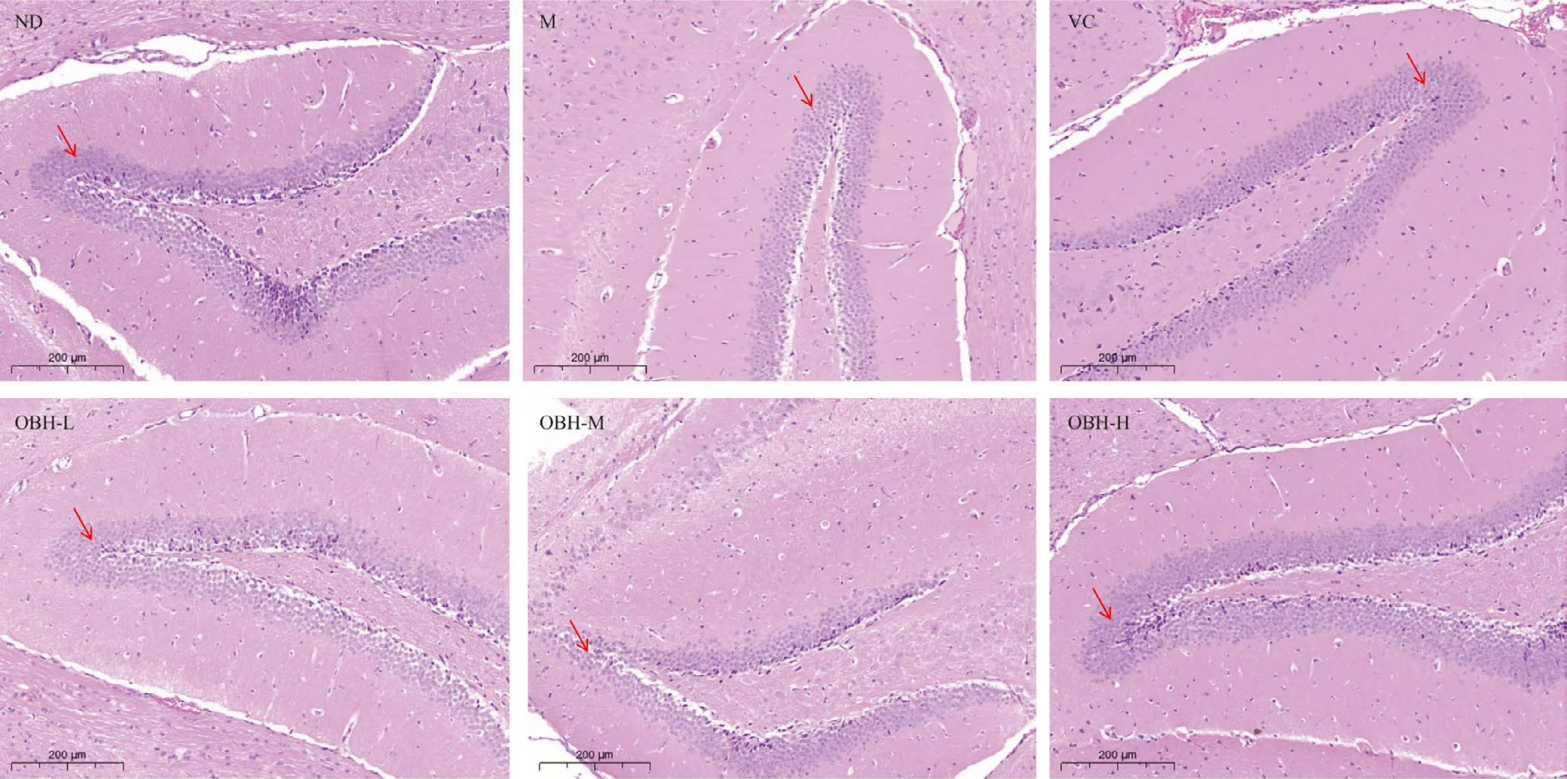
Effect of OBH intervention on brain histopathological alterations (H&E staining, scale bar 200 μm).

Figure 2 showed the brain histopathological analysis of diverse groups. The brain tissues in the normal mice showed a clear microstructure with tightly arranged pyramidal cells and clearly visible nucleoli with no abnormalities. On the other hand, the pathological features such as a reduced number of pyramidal cells and loosely arranged pyramidal cells could be seen within the brain tissues from the aging mice. Relative to the model group, VC and OBH high‐dose groups showed compactly arranged pyramidal cells with relatively high numbers. Therefore, pyramidal cells were partially lost in the brain tissues in the aging mice, which indicated the presence of adverse events that caused neuronal damage. Similarly, Guo et al. (2022) reported that the long‐time d‐gal injection induced brain damage, neuron damage or loss. OBH intervention alleviated this neuronal damage and protected from the d‐gal‐induced brain damage in a dose‐dependently manner.

### 3.5 Effect of OBH in Serum and Brain Antioxidant Enzyme Activities Within d‐Gal Aging Mice

Oxidative stress represents an imbalance of antioxidant defenses (enzymatic and nonenzymatic) in an organism, where abnormal body redox reactions generate excess free radicals like ROS to attacking biomolecules in the cell (Dedibegovic et al. 2020). There is a close relationship between oxidative stress and aging (Xiang et al. 2019). MDA, the oxidative toxicity product generated through lipid peroxidation, is a crucial indicator of cell membrane damage and organismal aging (Zhang, Yang, et al. 2020). In order to investigate the OBH antioxidant effects against in the d‐gal‐induced aging mice the MDA content was evaluated as well the T‐AOC, CAT, and GSH‐Px activities within the mice serum and brain.

#### 3.5.1 Effect of OBH in Serum Antioxidant Oxidase Activities

**FIGURE 3:**
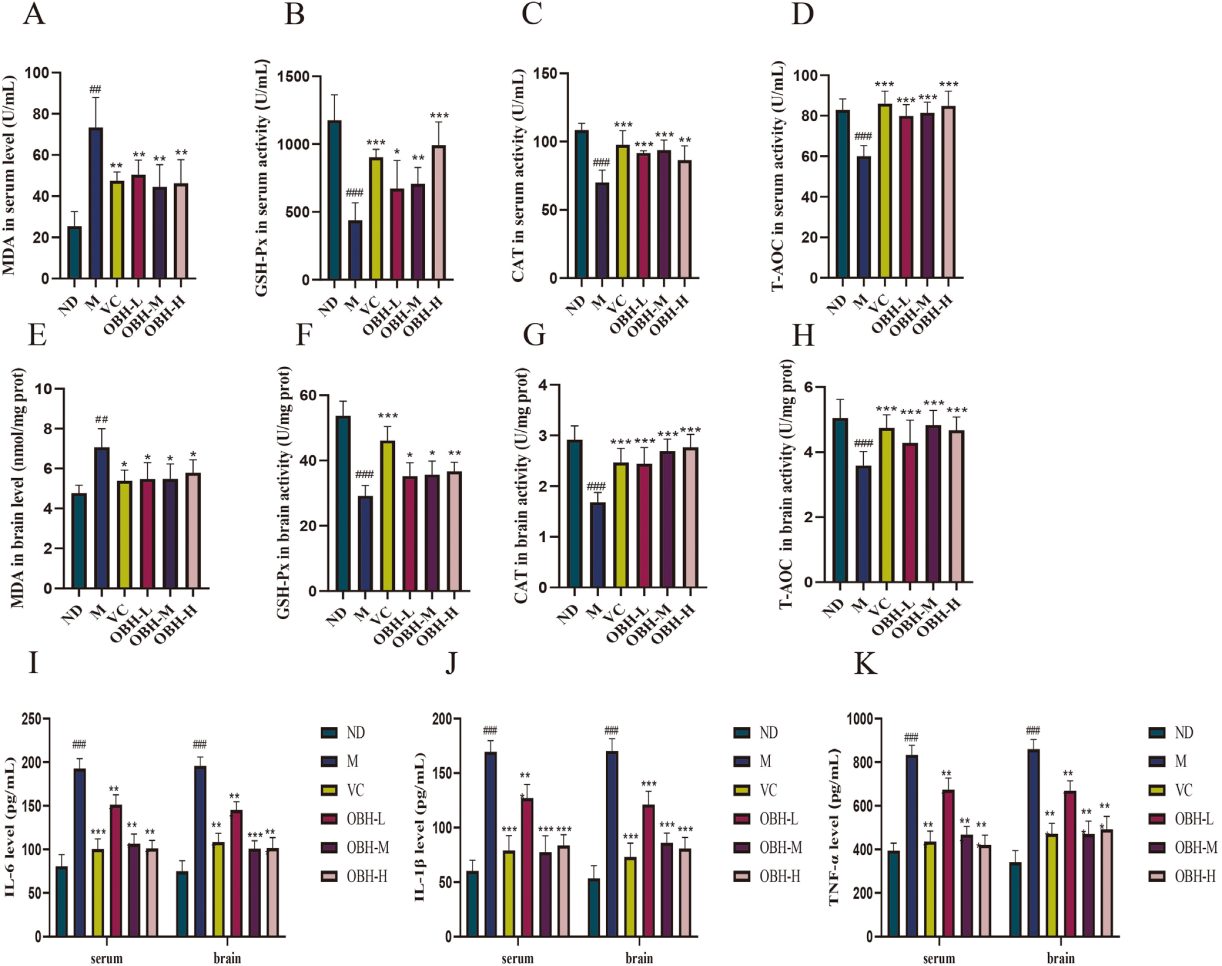
Effect of OBH on serum oxidative stress and antioxidant enzyme activities in mice (A–D). (A) MDA, (B) GSH‐Px, (C) CAT, and (D) T‐AOC. Effect of OBH on serum oxidative stress and antioxidant enzyme activities in mice (E–H). OBH inhibited d‐gal‐induced inflammatory activation. IL‐6, IL‐1β, and TNF‐α were detected in serum and brain tissue by ELISA (I–K). Data of each group were expressed as mean ± standard deviation (*n* = 6). Compared with the ND group, ^#^ *p* < 0.05, ^##^ *p* < 0.01, ^###^ *p* < 0.001; compared with the M group, *p < 0.05, **p < 0.01, ***p < 0.001.

Figure 3A–D shows the effect of OBH on the serum antioxidant enzyme activities. Compared to the normal group, the serum GSH‐Px, CAT, and T‐AOC activities of aging mice were significantly reduced (*p* < 0.01), whereas the MDA levels were significantly elevated (*p* < 0.01). Following interventions with VC and OBH, the GSH‐Px, CAT, and T‐AOC levels significantly elevated (*p* < 0.05), whereas MDA levels declined significantly (*p* < 0.05).

#### 3.5.2 Effect of OBH on the Brain Antioxidant Oxidase Activities

Figure 3E–H shows the role of OBH in antioxidase activities within the brain tissues. The differences in GSH‐Px, CAT, MDA, and T‐AOC levels of the model and normal groups were significantly different (*p* < 0.01), indicating that the d‐gal could cause brain damage. Compared to the model group, VC, OBH‐L, OBH‐M, and OBH‐H groups showed evidently reduced levels of MDA in the brain tissues (*p* < 0.01) but significantly increased levels of GSH‐Px, CAT, and T‐AOC (*p* < 0.05). An increase in these antioxidant enzyme activities was directly related to the dose of OBH, with the medium‐ and high‐dose groups and the VC group exhibiting a significant effect.

Therefore, the activities of GSH‐Px, CAT enzymes, and T‐AOC content elevated, and MDA content declined within the serum and brain tissues after OBH intervention compared to that in the model group. OBH inhibited oxidative damage by modulating antioxidant enzymes. Zhao et al. (2021) showed that fermented wheat germ significantly increased the antioxidant enzyme activities and reduced MDA levels within different liver, brain, and intestine tissues and serum.

### 3.6 Effect of OBH on Serum and Brain Inflammatory Response of d‐Gal Induced Aging Mice

Excessive production of ROS promotes inflammation, mediates the expression of pro‐inflammatory genes, and initiates a chronic inflammatory state. At the same time, the secretion of inflammatory cells leads to increased oxidative stress at the inflammatory site (Krzemińska et al. 2022; Michel et al. 2022). Aging disrupts the balance of proinflammatory and anti‐inflammatory cytokines, resulting in excessive production of proinflammatory cytokines like IL‐6, TNF‐α, and IL‐1β (Arnold et al. 2021). TNF‐α and IL‐6 can serve as biomarkers of aging, while IL‐1β is important for triggering age‐associated chronic inflammation (Baiocchi et al. 2021; Gonzalo‐Calvo et al. 2010).

In order to investigate the degree of inflammation, the brain and serum proinflammatory cytokines in each group were evaluated (Figure 3I–K). The serum and brain TNF‐α, IL‐6, and IL‐1β levels increased significantly in the model group compared to the normal group (*p* < 0.001) suggesting that d‐gal induced aging increased the secretion of multiple immune factors. OBH intervention significantly reduced the levels of TNF‐α, IL‐6, and IL‐1β in the serum and brain in the aging mice compared to those of the model group (*p* < 0.001). Consistently, Zou et al. (2023) showed that *Codonopsis pilosula* pectic polysaccharides reduced these factors within the intestine and liver, which delayed aging by attenuating the inflammatory response. Wu et al. (2022) demonstrated that whey protein hydrolysate reduced their levels in the serum of aging mice as well. Therefore, OBH intervention can reduce inflammatory cytokine secretion and effectively alleviate inflammation in the aging mice.

### 3.7 Effect of OBH on Gut Microbiota in d‐Gal Aging Mice

There is a close relationship between gut microbiota and oxidative stress, and the body oxidative stress levels are affected by gut microbiota through the synthesis of metabolites, regulating antioxidant enzymes, and maintaining intestinal homeostasis, whereas oxidative stress may affect gut microbiota by promoting dysbiosis. (Sun et al. 2024; Shandilya et al. 2022; Singh et al. 2023). Human aging is related to the gut microbiota, and the regulation of gut microbes may be able to intervene in aging (Du et al. 2021). Changes in the gut microbiota of ND, M and OBH‐H intervention group mice were explored by 16S rRNA high‐throughput gene sequencing.

**FIGURE 4:**
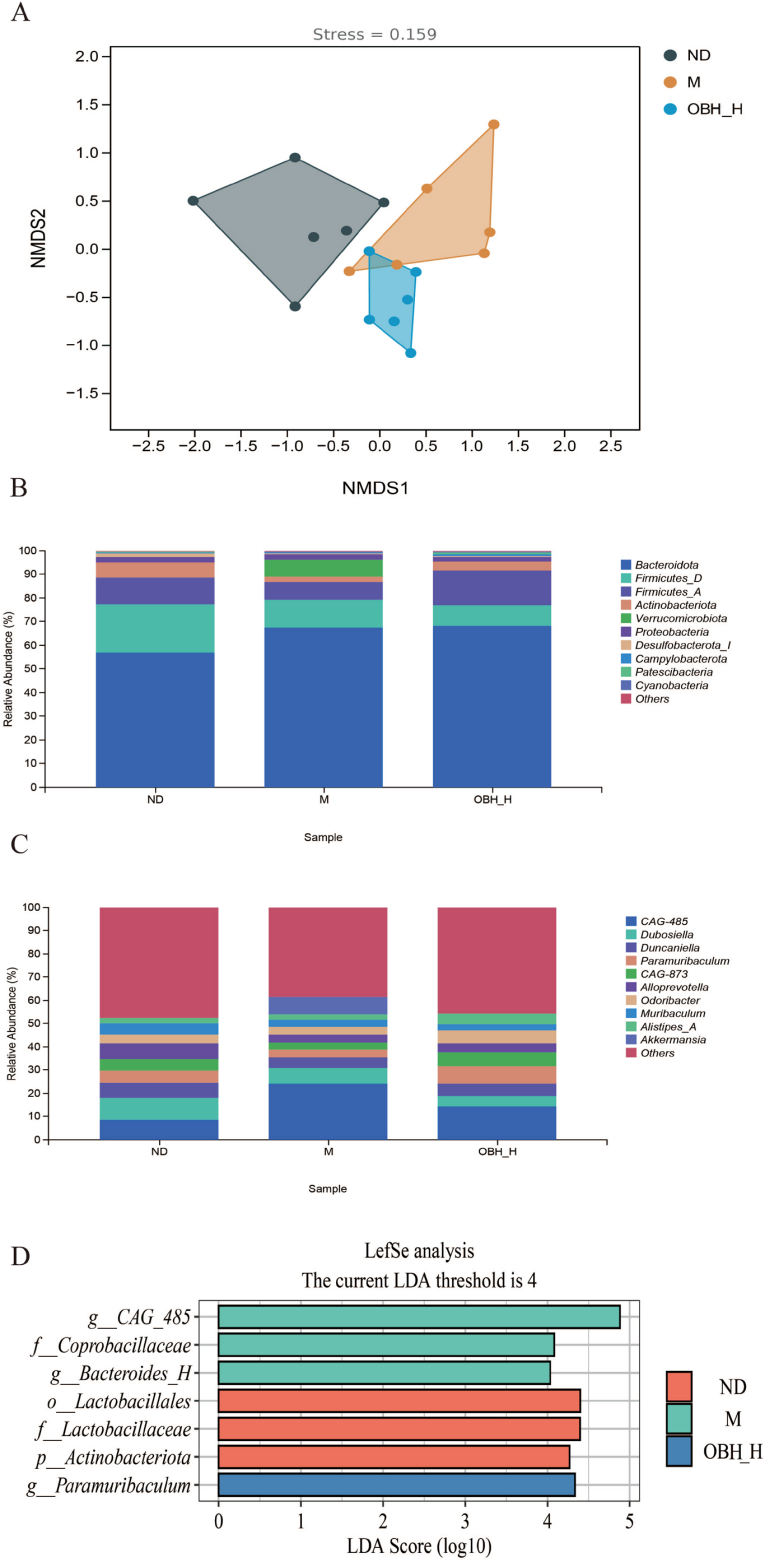
Effect of OBH on the gut microbiota of d‐gal aging mice. Nonmetric multidimensional scaling (NMDS, Stress = 0.159) score plot under Bray‐Curtis for samples with stress below 0.2, where the degree of bias between samples is accurately reflected (A). Distribution of gut microbiota at the phylum level (B) and genus level (C). LDA score distribution histograms showing microorganisms with significant differences in abundance in different groups (D).

Based on nonmetric multidimensional scaling (NMDS), the microbial composition segregated from ND and M groups was obviously significantly different, and the OBH intervention modulated the structure and composition of gut microbiota in the aging mice (Figure 4A). Gut microbial abundance was further analyzed at phylum and genus levels. At a phylum level (Figure 4B), those major gut microorganisms in the mice of ND, M, and OBH groups consisted of *Bacteroidota*, *Firmicutes*, *Actinobacteria*, and *Proteobacteria*, with *Bacteroidota* and *Firmicutes* representing 50.96% and 19.22% or more. It was indicated that the Firmicutes‐to‐Bacteroides (F/B) ratios were closely associated with aging (Liu et al. 2023), and they were significantly altered among the three groups. The F/B ratio in the ND group was 0.65, which was increased from 0.30 to 0.43 in the M group after the OBH intervention. This is consistent with the previously reported results (Zhang, Yang, et al. 2020; Zhang, He, et al. 2020). In addition, the relative abundances of *Verrucomicrobiota* were significantly reduced in the OBH‐H group compared to the M group. At a genus level (Figure 4C), the gut microbiota in the mice in each group included *CAG‐485*, *Dubosiella*, *Duncaniella*, *Paramuribaculum*, *CAG‐873*, *Alloprevotella*, *Odoribacter*, *Muribaculum*, and *Alistipes_A*. Compared to the ND group, the abundances of *CAG‐485* were significantly increased and those of *Dubosiella*, *Duncaniella*, *Paramuribaculum*, *CAG‐873*, *Odoribacter*, and *Alistipes_A* were significantly decreased in the M group. OBH intervention reduced the relative abundances of *CAG‐485* and elevated those of *Duncaniella*, *Paramuribaculum*, *CAG‐873*, *Odoribacter*, and *Alistipes_A*. Additionally, LEfSe analysis of variance was performed, showing that the ND group was enriched in *o_Lactobacillales*, *f_Lactobacillaceae*, and *p_Actinobacteriota*; the M group was enriched in *g_CAG_485*, *f_Coprobacillaceae*, and *g_Bacteroidesg_H*; and the OBH‐H intervention group was enriched in *g_Paramuribaculum* (Figure 4D).

### 3.8 Analyze of OBH on NonTargeted Metabolism Within the Brain Tissues in d‐Gal Aging Mice

**FIGURE 5:**
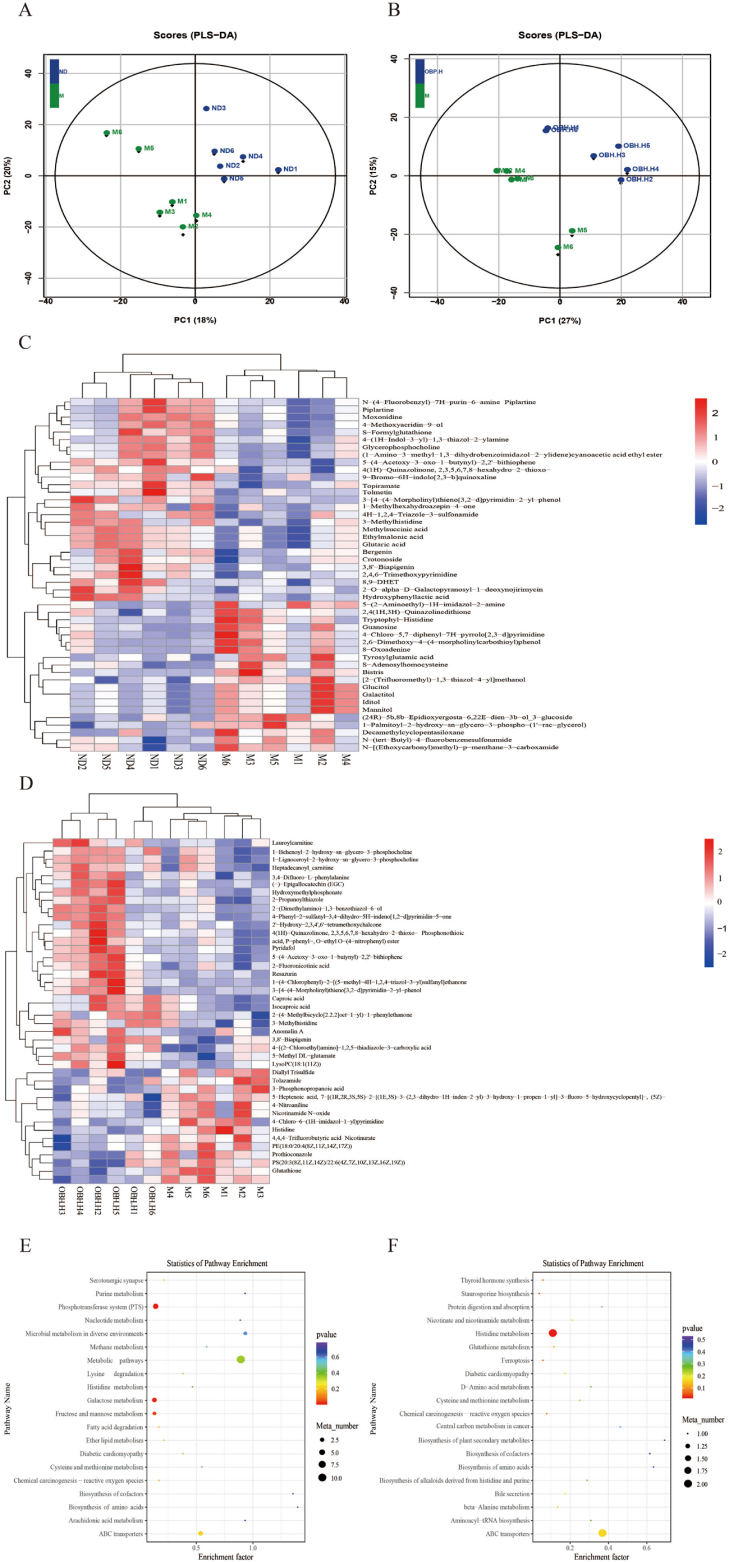
OBH altered brain metabolism in senescent mice (*n* = 6). Partial least squares discriminant analysis (PLS‐DA) for ND and M groups (A) and partial least squares discriminant analysis (PLS‐DA) for M and OBH‐H intervention groups (B). Cluster analysis of metabolites in both ND and M groups to determine differences between the two groups (C), and cluster analysis of metabolites in both M and OBH‐H intervention groups (D). Each row in the figure represents a metabolite. Each column represents one sample. The color indicates the relative expression of the metabolite in the group; red indicates high metabolite expression and blue indicates low metabolite expression. Differential metabolite pathway enrichment in two groups, the ND and M groups (E), and in two groups, the M and OBH‐H intervention groups (F).

The brain has been considered a critical complex organ with important structures and functions in mammals, and it exerts its functions through neurochemistry (Ding et al. 2021). However, studies on complex brain metabolome together with the alterations in aging are scarce. Nontargeted metabolite analysis of different mouse groups not only helps identify specific metabolites but also provides insights into the OBH inhibitory mechanism of oxidative stress. This study explored how OBH administration affected the nontargeted metabolome of the brain tissues from d‐gal‐induced aging mice. In total, 1336 metabolites were identified, most of which were amino acids, glycoconjugates, fatty acids, nucleosides, and purines. After supervised partial least squares discriminant analysis (PLS‐DA), ND and M groups and M and OBH‐H intervention groups were evidently separated (Figure 5A,B).

Additionally, in order to evaluate differential metabolites within the brain tissues in the d‐gal aging mice via OBH gavage, the metabolites in the ND and M groups (Figure 5C) and the M and OBH‐H groups (Figure 5D) were subjected to cluster analysis using heatmaps (*p* < 0.05, VIP > 1 and FC > 1). Two distinct clusters were formed among these different metabolites, with the most metabolites in the ND and M groups and M and OBH‐H groups being in different branches. This result suggests that a successful modeling and OBH gavage can alter brain metabolites in the aging mice. As demonstrated in Figure 5C, compared to the ND group, the glycerophosphocholine and S‐formylglutathione contents were significantly decreased within the brain tissues of the M group (*p* < 0.05). In contrast, iditol, mannitol, galactitol, glucitol, guanosine, and S‐adenosylhomocysteine contents were significantly elevated (*p* < 0.05). Furthermore, as shown in Figure 5D, the levels of S‐adenosylhomocysteine, prothioconazole, and PS (20:3(8Z, 11Z, 14Z)/22:6(4Z, 7Z, 10Z, 13Z, 16Z, 19Z)) were significantly decreased within the brain tissues in the mice of the OBH‐H group (*p* < 0.05) compared to those of the M group. However, 1‐lysophosphatidylcholine (LysoPC, 18:1(11Z)), phosphatidylcholine (PC, 33:2), phosphoethanolamine (PE, 18:0/20:5 (5Z, 8Z, 11Z, 14Z, 17Z)), glycerophosphocholine, and (−)‐ epigallocatechin (EGC) levels were obviously elevated (*p* < 0.05). This study used KEGG topology to analyze the corresponding metabolic pathways enrichment, and it was observed that galactose metabolism, phosphotransferase system, glycerophospholipid metabolism, fructose and mannose metabolism, arachidonic acid metabolism, and cysteine and methionine metabolism were primarily consisted of in the ND and M groups (Figure 5E). On the other hand, glycerophospholipid metabolism, cysteine and methionine metabolism, chemical carcinogenesis‐ROS, and histidine metabolism were primarily consisted of in the M and OBH‐H groups (Figure 5F). These trends suggest that d‐gal‐induced aging is closely related to glucose metabolism, cysteine and methionine metabolism, and glycerophospholipid metabolism and that OBH can alleviate d‐gal aging‐induced metabolic disorders.

Lipid metabolism disorders can lead to brain aging and cognitive dysfunction (Hu et al. 2022). A potential relationship between brain aging and lipid metabolism was found in this study. Experiments revealed that PE (18:0/20:5 (5Z, 8Z, 11Z, 14Z, 17Z)), LysoPC (18:1 (11Z)), PC (33:2), and glycerophosphocholine, as potential biomarkers, are associated with glycerophospholipid metabolism. LysoPC (18:1(11Z)), PC (33:2), PE (18:0/20:5(5Z, 8Z, 11Z, 14Z, 17Z)), and glycerophosphocholine contents declined within d‐gal‐induced aging mice compared to ND mice. In contrast, their contents were increased in mice after OBH‐H administration. This showed that OBH can effectively improve glycerophospholipid metabolism disorders in the d‐gal aging mice. As reported previously, researchers revealed a significant correlation between PS (20:3(8Z, 11Z, 14Z)/22:6(4Z, 7Z, 10Z, 13Z, 16Z, 19Z)) and phospholipid metabolism disorders (Meng et al. 2023). Prothioconazole results in triglyceride accumulation, changing glycolipid metabolism‐associated gene and metabolite expression within mice, which affects glycolysis and pyruvate metabolic pathways (Tian et al. 2021). Both prothioconazole and PS (20:3(8Z, 11Z, 14Z)/22:6(4Z, 7Z, 10Z, 13Z, 16Z, 19Z)) levels were decreased in the OBH intervention group. Methionine produces methyl donor S‐adenosylmethionine, and the latter is then transformed to S‐adenosylhomocysteine through methylation, accumulating during aging (You et al. 2023). Compared to the ND group, S‐adenosylhomocysteine expression in the M group was increased but was decreased after OBH administration. This result indicates that OBH significantly intervention methionine and cysteine metabolism. This conforms to findings reported by Parkhitko et al. (2016), suggesting that downregulating tissue, specifically S‐adenosylhomocysteine prolongs the healthy period and lifespan of Drosophila. EGC, as a potential antioxidant with the ability to effectively neutralize ROS, can pass through the blood–brain barrier into the brain parenchyma, promote neuronal differentiation, and inhibit brain aging (Ambigaipalan et al. 2019). The OBH‐H intervention group significantly increased the content of EGC in comparison to the aging model group. Therefore, OBH could improve aging by altering metabolism in the d‐gal‐induced aging mice.

### 3.9 Correlation of Gut Microbiota With Differential Metabolites

**FIGURE 6:**
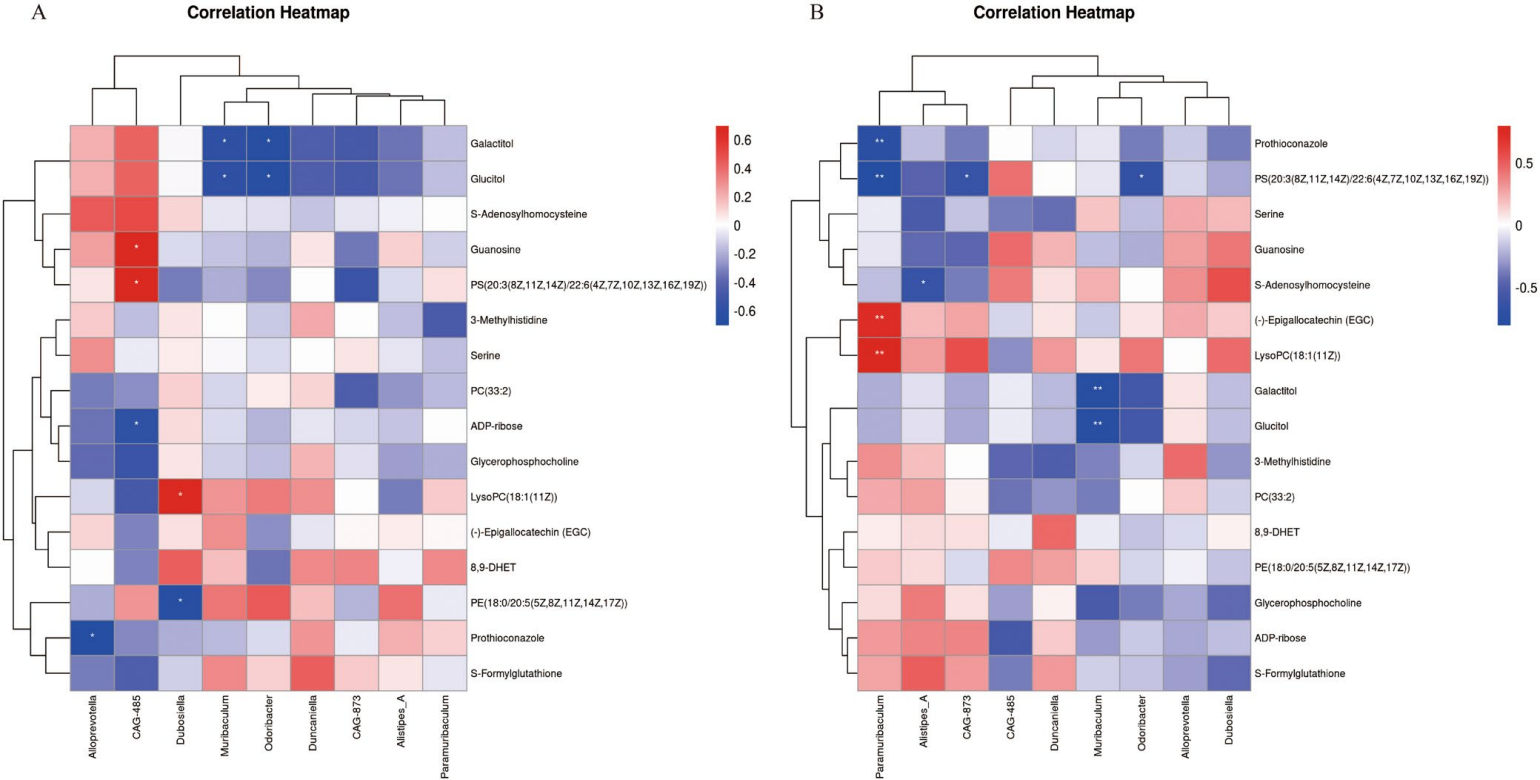
Correlation analysis of gut microbiota and differential metabolites in ND and M groups (A), and correlation analysis of gut microbiota and differential metabolites in M and OBH‐H intervention groups (B). Positive correlations are indicated in red, while negative correlations are shown in blue, with significance levels marked as *p < 0.05 and **p < 0.01.

The association between differential metabolites and gut microbiota in the ND and M groups and M and OBH‐H groups was explored through Spearman's correlation analysis. The gut microbiota showed significant correlations with different metabolites. As presented in Figure 6A, *CAG‐485* was positively correlated with the content of PS(20:3(8Z, 11Z, 14Z)/22:6(4Z, 7Z, 10Z, 13Z, 16Z, 19Z)) and guanosine, but negatively correlated with the content of ADP‐ribose. *Dubosiella* was positively correlated with LysoPC (18:1(11Z)) correlation. *Odoribacter* was negatively correlated with glucitol and galactitol. As shown in Figure 6B, *Paramuribaculum* was positively correlated with LysoPC (18:1(11Z)) and (−)‐Epigallocatechin (EGC) levels, but negatively correlated with prothioconazole and PS(20:3(8Z, 11Z, 14Z)/22:6(4Z, 7Z, 10Z, 13Z, 16Z, 19Z)) levels. PS(20:3(8Z, 11Z, 14Z)/22:6(4Z, 7Z, 10Z, 13Z, 16Z, 19Z)) content was negatively correlated with *CAG‐873* and *Odoribacter*. Based on the above findings, gut microbiota is closely related to cysteine and methionine metabolism, glycerophospholipid metabolism, the chemical carcinogen, and ROS, and they work together to improve aging.

## 4 Conclusion

In this study, we obtained four fractions (molecular weights > 10, 3–10, 1–3, and < 1 kDa) were obtained via enzymatic hydrolysis and ultrafiltration of the proteins extracted from oat bran. Among them, fraction F1 (> 10 kDa, OBH) possesses the highest antioxidant activity and exerts protection against brain damage in the d‐gal‐induced aging mice. Furthermore, it increases serum and brain antioxidase activities in the aging mice and decreases the content of MDA, a lipid oxidation product, and the immune factors in the serum and brain. OBH can regulate gut microbes composition. Further analysis of brain tissue metabolites revealed the disruption of glucolipid metabolism during the aging process. OBH can regulate glycerophospholipid metabolism and methionine and cysteine metabolism and postpone the aging process of the d‐gal‐induced mice. Our findings provide a theoretical foundation to comprehensively investigate OBH as a potential antioxidant candidate and to entirely utilize OBH can alleviate oxidative stress and inflammation in vivo. However, the structural composition of OBH and the isolation and identification of hydrolysates, as well as the mechanism of oxidative stress in vivo, still need to be further studied, we will quantify it using standard that can be used for targeted metabolomics analysis.

## Author Contributions

**Haoyuan Ma:** conceptualization (equal), data curation (equal), methodology (equal), writing – original draft (equal). **Rui Wang:** data curation (equal), investigation (equal), methodology (equal), software (equal). **Minjun Sun:** data curation (equal), formal analysis (equal). **Sarina Ma:** conceptualization (equal), funding acquisition (equal), supervision (equal), writing – review and editing (equal). **Meili Zhang:** funding acquisition (equal), supervision (equal), writing – review and editing (equal).

## Conflicts of Interest

The authors declare no conflicts of interest.

## Supporting information


Appendix S1

## Data Availability

All relevant data are included within the manuscript. Any inquiries concerning the findings of this study may be directed to the corresponding author upon request.
