# Supplementary material for: Oat Bran Hydrolysates Alleviate Oxidative Stress and Inflammation in d‐Galactose‐Induced Aging Mice
Source: Food Sci Nutr. 2025 Jun 17;13(6):e70433. doi: 10.1002/fsn3.70433 (PMC12171632; doi:10.1002/fsn3.70433)
Supplement: Supplementary file 1 — Appendix S1 [file FSN3-13-e70433-s001.docx]

**Appendix**


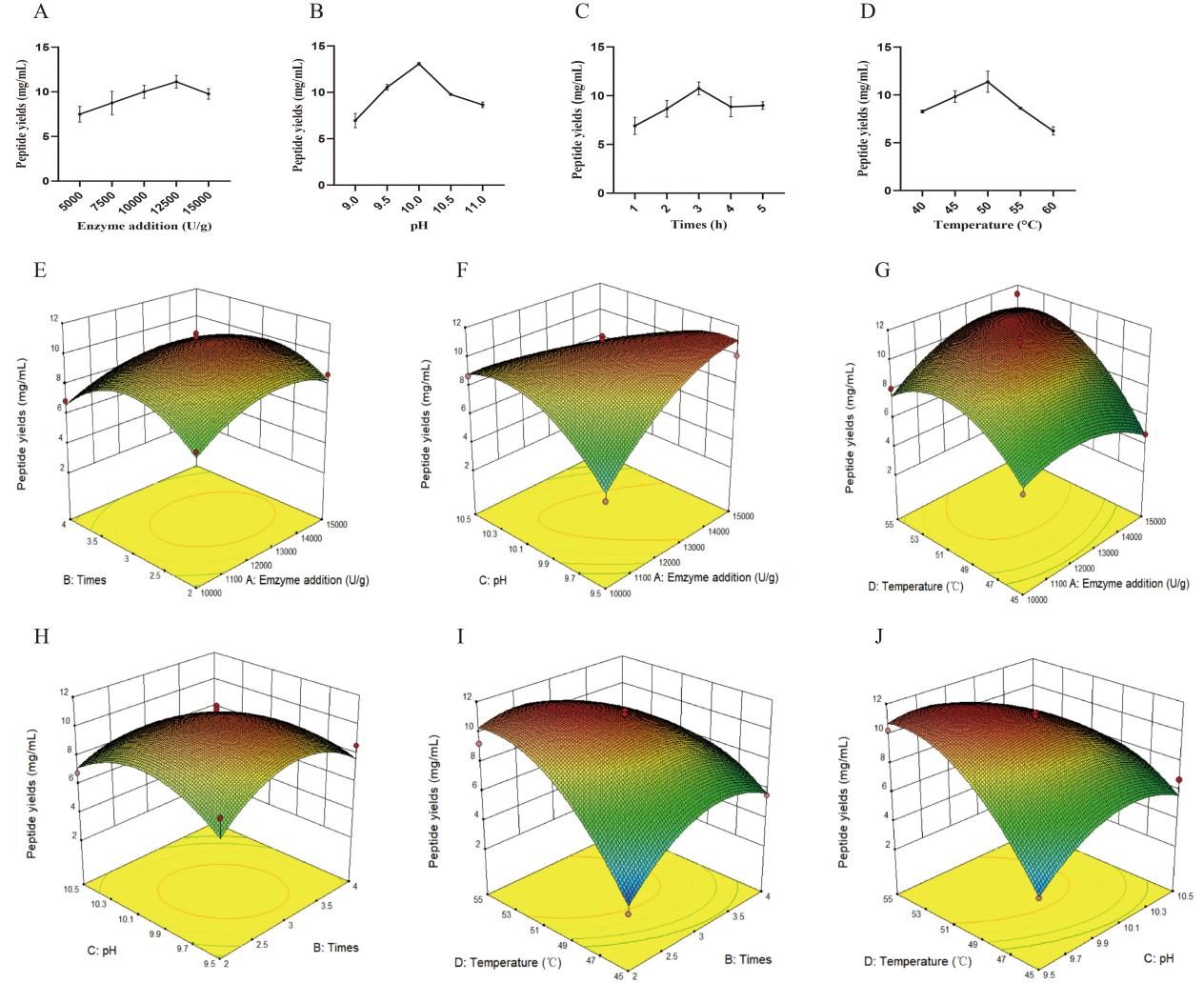


**Fig. S1** Optimisation of OBP enzymatic process.The effect of enzyme addition, pH, time and temperature on the enzyme digestion at 2% substrate mass concentration (A-D). 3D surface of different factors (E-J).

**Table S1.** Response surface test design and results

| Run | *A* Emzyme addition (U/g) | *B* Times (h) | *C* pH | *D* Temperature (℃) | *Y* Peptide yields (mg/mL) |
| --- | --- | --- | --- | --- | --- |
| 1 | 12 500 | 3 | 10.0 | 50 | 11.34 |
| 2 | 15 000 | 3 | 9.5 | 50 | 9.96 |
| 3 | 12 500 | 3 | 9.5 | 55 | 10.25 |
| 4 | 10 000 | 4 | 10.0 | 50 | 6.90 |
| 5 | 15 000 | 3 | 10.5 | 50 | 3.99 |
| 6 | 12 500 | 3 | 9.5 | 45 | 3.48 |
| 7 | 12 500 | 3 | 10.5 | 55 | 6.68 |
| 8 | 12 500 | 4 | 10.5 | 50 | 3.84 |
| 9 | 12 500 | 4 | 9.5 | 50 | 8.57 |
| 10 | 12 500 | 4 | 10.0 | 55 | 5.30 |
| 11 | 10 000 | 3 | 9.5 | 50 | 4.57 |
| 12 | 15 000 | 3 | 10.0 | 45 | 4.72 |
| 13 | 12 500 | 2 | 9.5 | 50 | 7.99 |
| 14 | 10 000 | 3 | 10.0 | 45 | 5.37 |
| 15 | 12 500 | 2 | 10.0 | 45 | 2.50 |
| 16 | 10 000 | 3 | 10.0 | 55 | 8.06 |
| 17 | 10 000 | 3 | 10.5 | 50 | 8.72 |
| 18 | 12 500 | 3 | 10.0 | 50 | 10.47 |
| 19 | 12 500 | 2 | 10.5 | 50 | 6.83 |
| 20 | 15 000 | 2 | 10.0 | 50 | 8.65 |
| 21 | 12 500 | 3 | 10.0 | 50 | 11.12 |
| 22 | 12 500 | 3 | 10.5 | 45 | 6.75 |
| 23 | 12 500 | 3 | 10.0 | 50 | 10.54 |
| 24 | 15 000 | 3 | 10.0 | 55 | 11.34 |
| 25 | 12 500 | 3 | 10.0 | 50 | 10.10 |
| 26 | 15 000 | 4 | 10.0 | 50 | 7.05 |
| 27 | 10 000 | 2 | 10.0 | 50 | 7.12 |
| 28 | 12 500 | 2 | 10.0 | 55 | 9.26 |
| 29 | 12 500 | 4 | 10.0 | 45 | 5.59 |

**Table S2.** ANOVA for Response Surface Quadratic model

| Source | Sum of Squares | df | Mean Square | F Value | p-value Prob > F |  |
| --- | --- | --- | --- | --- | --- | --- |
| Model | 176.30 | 14 | 12.59 | 15.64 | < 0.0001 | *** |
| *A-*Emzyme addition | 2.04 | 1 | 2.04 | 2.53 | 0.1337 |  |
| *B-*Times | 2.13 | 1 | 2.13 | 2.65 | 0.1261 |  |
| *C-*pH | 5.35 | 1 | 5.35 | 6.64 | 0.0219 | *** |
| *D-*Temperature | 42.26 | 1 | 42.26 | 52.48 | < 0.0001 | ***** |
| *AB* | 0.48 | 1 | 0.48 | 0.59 | 0.4538 |  |
| *AC* | 25.59 | 1 | 25.59 | 31.77 | < 0.0001 | ***** |
| *AD* | 3.86 | 1 | 3.86 | 4.8 | 0.046 | *** |
| *BC* | 3.18 | 1 | 3.18 | 3.95 | 0.0668 |  |
| *BD* | 12.58 | 1 | 12.58 | 15.63 | 0.0014 | **** |
| *CD* | 11.68 | 1 | 11.68 | 14.51 | 0.0019 | **** |
| *A*2 | 12.04 | 1 | 12.04 | 14.96 | 0.0017 | **** |
| *B*2 | 32.04 | 1 | 32.04 | 39.79 | < 0.0001 | ***** |
| *C*2 | 24.98 | 1 | 24.98 | 31.02 | < 0.0001 | ***** |
| *D*2 | 33.08 | 1 | 33.08 | 41.08 | < 0.0001 | ***** |
| Residual | 11.27 | 14 | 0.81 |  |  |  |
| *Lack of Fit* | 10.25 | 10 | 1.03 | 4.01 | 0.0966 |  |
| *Pure Error* | 1.02 | 4 | 0.26 |  |  |  |
| Cor Total | 187.57 | 28 |  |  |  |  |
| R-Squared=0.9399, Adj R-Squared=0.8798 | | | | | | |
